# A Freshwater Streptomyces, Isolated from Tyume River, Produces a Predominantly Extracellular Glycoprotein Bioflocculant

**DOI:** 10.3390/ijms13078679

**Published:** 2012-07-13

**Authors:** Uchechukwu U. Nwodo, Mayowa O. Agunbiade, Ezekiel Green, Leonard V. Mabinya, Anthony I. Okoh

**Affiliations:** Applied and Environmental Microbiology Research Group (AEMREG), Department of Biochemistry and Microbiology, University of Fort Hare, Private Bag X1314, Alice 5700, South Africa; E-Mails: unwodo@ufh.ac.za (U.U.N.); egreen@ufh.ac.za (E.G.); lmabinya@ufh.ac.za (L.V.M.); mayorlala@gmail.com (M.O.A.)

**Keywords:** bioflocculant, culture conditions, streptomyces, chemical composition, pyrolysis, SEM imaging

## Abstract

We evaluated bioflocculant production by a freshwater actinobacteria whose 16S rDNA nucleotide sequence was deposited in GenBank as *Streptomyces* sp. Gansen (accession number HQ537129). Optimum culture conditions for bioflocculant production were an initial medium pH of 6.8, incubation temperature of 30 °C, agitation speed of 160 rpm and an inoculum size of 2% (v/v) of cell density 1.5 × 10^8^ cfu/mL. The carbon, nitrogen and cation sources for optimum bioflocculant production were glucose (89% flocculating activity), ammonium sulfate (76% flocculating activity) and MgCl_2_. Bioflocculant pyrolysis showed three step decomposition indicative of three components while chemical analyses showed 78% carbohydrate and 22% protein (wt/wt). The mass ratio of neutral sugar, amino sugar and uronic acids was 4.6:2.4:3. FTIR spectrometry indicated the presence of carboxyl, hydroxyl and amino groups, typical for heteropolysaccharide. The bioflocculant showed a lattice structure as seen by SEM imaging. Its high flocculation activity suggests its suitability for industrial applicability.

## 1. Introduction

Flocculants have diverse properties which inform their uses in flocculation technology. However, the ability to floc out suspended solutes from solvents indicates their suitability for use in water processing, particularly municipal and wastewater treatment [1–4].

Flocculants are categorized into three groups, namely the inorganic flocculants which includes salts of aluminum (aluminum sulfate and poly-aluminum chloride); the organic synthetic polymeric flocculants (polyacrylamide derivatives and polyethylene imines), and the natural occurring flocculants of microbial origin, termed bioflocculant, which includes chitosan and sodium alginate [5–7]. The inorganic and organic synthetic polymeric flocculants are widely used in industrial processes as they possess high efficiency of flocculation and are inexpensive [6,8]; however associated demerits have been colossal and it includes deleterious health problems such as cancer and neuro-toxicity in the case of polyacrylamide derivatives and polyethylene imines and Alzheimer’s disease with respect to salts of aluminum [9–11]. In addition, organic synthetic polymeric flocculants constitute a nuisance to the environmental because they are non-degradable and degradation attempt leaves behind monomeric units which could wash into underground waters [12].

Bioflocculants, on the other hand, are safe, biodegradable and lack secondary pollution from degradation intermediates. The enormous advantages associated with bioflocculants have attracted considerable scientific attention [5,13,14]. However, high production cost and low flocculation efficiency has been a limiting factor for industrial application of microbial-produced flocculants. As a result, there is a continual exploration for microbes with high bioflocculant yield, high flocculation efficiency and minimal production cost.

Though members of the actinobacteria group have been well documented as major sources of secondary metabolites of economic importance (two-thirds occurring as antibiotics), very scanty information exists implicating them in biofloculant production. In this report, we evaluate the bioflocculant production by a freshwater Streptomyces isolated from Tyume river in South Africa. Culture conditions were manipulated to optimize bioflocculant yield and the bioflocculant also characterized for novelty.

## 2. Results and Discussion

### 2.1. Actinobacteria Identification

The polymerase chain reaction (PCR) amplification of the 16S rRNA gene of the gram positive bacteria resulted in a PCR product of the expected size (1.5 kb). Basic Local Alignment Search Tool (BLAST) analysis of the nucleotide sequence of the 16S rDNA showed the bacteria to have 99% similarity to that of *Streptomyces* sp. MEC01 and the nucleotide sequence was deposited in GenBank as *Streptomyces* sp. Gansen with accession number HQ 537129.

### 2.2. The Effect of Starter Culture Density on Bioflocculant Activity

**Figure 1:**
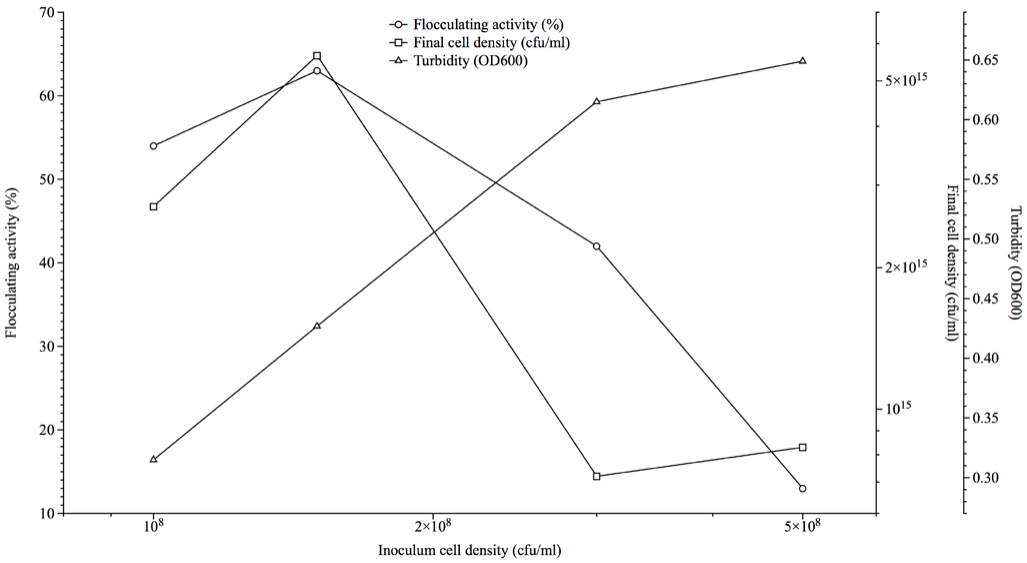
Effect of inoculum cell density on bioflocculant production by *Streptomyces* sp. Gansen (HQ537129).

Inocula cell densities corresponding to 1.0, 1.5, 3.0 and 5.0 (×10^8^ cfu/mL) used as 2% of the fermentation media resulted in flocculation activities of 54%, 63%, 42% and 13% respectively after 72 h, while corresponding cell densities measured at 600nm optical densities (OD_600_), varied from 0.427 to 3.315 and viable count ranged 27–83 × 10^15^ (cfu/mL) (Figure 1). The viable count method was espoused for the kinetics studies as it showed more accuracy towards measuring cell density at the point of flocculation activity measurement.

Cost effectiveness in the industrial production of valuable microbial products via fermentation includes optimized inocula cell density to nutrient ratio for synchronous cultures to exhibit maximal potentials. Mullins and NeSmith as well as Gibbons and Westby similarly demonstrated the phenomenon of culture to nutrient ratio optimization in the production of ethanol [15,16].

### 2.3. Effect of Culture Conditions on Bioflocculant Production

**Figure 2:**
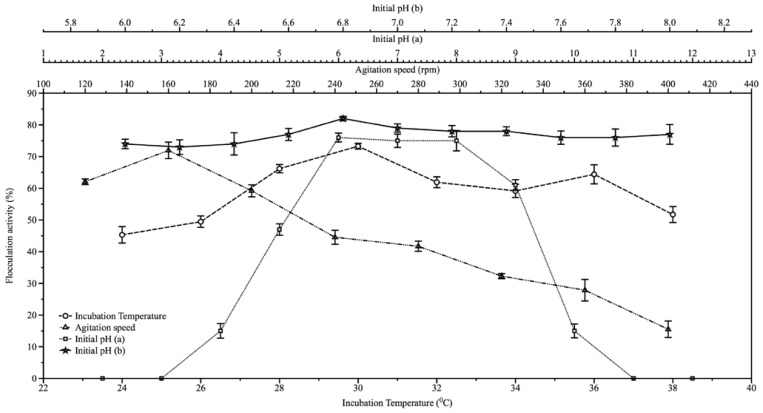
The effect of initial fermentation pH, incubation temperature and agitation speed on the production of bioflocculant by *Streptomyces* sp. Gansen (HQ537129).

The result of the effect of initial medium pH on bioflocculant production is shown in Figure 2. The highest flocculation activities were initially obtained at pH range of between 6 and 8 (Figure 2) with flocculation activities of 76% and 75% respectively. However, on further evaluation of pH regimes of between 6 and 8, the highest flocculation activity (82%) was recorded at pH 6.8 (Figure 2). Also, temperature optimum was observed to be 30 °C with flocculation activity of about 73.3% (Figure 2); while agitation speed of 160 rpm was optimum for the bioflocculant production resulting in flocculation activity of about 72%. Subsequent increase in agitation speed (200 to 400 rpm) resulted in decrease in flocculation activities (Figure 2). The initial fermentation pH of near neutrality, moderate agitation speed and incubation temperature requirements of the actinobactaria for bioflocculant production appear to be related to its ecological niche being of freshwater milieu where some of the attributes measured might be moderate. Bioflocculant production at neutral pH was similarly reported by Mabinya *et al*. [17]. Consequently, production costs attributed to physicochemical parameters would be expected to be lower in the event of using *Streptomyces* sp. Gansen (HQ537129) for bioflocculant production.

### 2.4. Effect of Nutritional Factors on Bioflocculant Production

Glucose served as best carbon source resulting in optimum flocculation activity of about 89.3% and bioflocculant yield of 3.37 g/L. Fructose followed with flocculating activity of about 86.4% and yield of 3.4 g/L, while starch showed least flocculation activity (38.9%) with a yield of 1.52 g/L (Table 1). Also, the inorganic nitrogen source (ammonium sulfate) resulted in optimal production of the bioflocculant with flocculation activity of about 76.3% and bioflocculant yield of 3.26 g/L; while magnesium chloride was the best cation source with flocculation activity of about 73.2% with yield of 3.39 g/L. The choice of cheap nitrogen and cation sources (which are abundant as mineral salts in nature) for bioflocculant production is remarkable from the perspective of the economics of industrial scale production. However, preference of simple sugars, like glucose, fructose and sucrose has been reported to be favorable for bioflocculant production by *Corynebacterium glutamicum* [13,14]. In the same vein, Gonzales and Hu [18] reported the stimulation of *Flavobacterium* sp. by Ca^2+^, Mn^2+^, and Ba^2+^, in bioflocculant production while Takagi and Kadowaki [19] similarly, reported enhanced cell growth and flocculating activity of *Paecilomyces* sp. stimulated by Ca^2+^.

### 2.5. Kinetics of Bioflocculant Production

**Figure 3:**
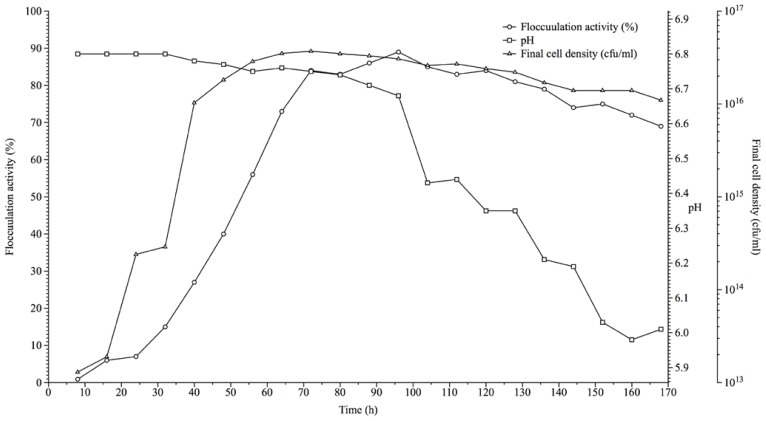
The growth curve of *Streptomyces* sp. Gansen (HQ537129) over a period of time with respect to bioflocculant production.

A 2% (v/v) starter culture of cell density 1.5 × 10^8^ (cfu/mL) used for bioflocculant production over a duration of seven (7) days showed growth curve as seen in Figure 3. There was a steep increase in the flocculation activity between 24 h (7% activity) to 72 h (84% activity) of fermentation and this coincides with the logarithmic growth phase of the actinobacteria as cell density ranged 29 × 10^13^ to 37 × 10^15^ cfu/mL respectively. However, maximum flocculation activity (89%) was achieved at 96 h of fermentation after which it slowly decreased to 85% and progressed to 81% at 104 h and 128 h respectively, depicting the stationary and early decline phase of cell growth (Figure 3). At 128 h, there was a steep decline in bacterial cell count and a gradual but steady decline in flocculation activity. The optimal flocculation activity shown at the logarithmic phase of growth is an indication that biosynthetic processes were responsible for production of the bioflocculant in corroboration of the report Lu *et al*. [20].

### 2.6. Effects of Cations and pH on Flocculation Activity of Purified Bioflocculant

The effects of monovalent, divalent and trivalent cations on flocculation activity of purified bioflocculant (Table 2), showed that divalent cations aided flocculation better than monovalent and trivalent cations. For example, the following flocculation activities were observed: 87% (Ca^2+^); 76% (Mg^2+^); and 62% (Mn^2+^) respectively, against the partial purified bioflocculant (PPB), and 72% (Ca^2+^); 57% (Mg^2+^); and 54% (Mn^2+^) against the cetylpyridinium chloride (CPC) purified bioflocculant (CPB) respectively. The role of cations in the mediation of flocculation activity by bioflocculants has been similarly documented [21,22]. However, attempt to remove metal ions by dialysis as conducted by Gong *et al*. [21] was not compare to non dialyzed fraction as has been shown in this study. On the other hand, the monovalent and trivalent cations did not show much variation on their influence on flocculation activity although they showed activity much lower than the divalent cations. However, considering that these cations have different binding capacities, it could be that the divalent metals showed more availability of binding sites and thus more anchorage to the bioflocculants and consequently yielding higher flocculation activity.

Both CPB and PPB achieved optimum flocculation at pH 7 (flocculation activities of 88% and 77% respectively) (Table 2). This is not surprising as the effects of pH and cations are inextricably linked in flocculation process. This is so because at low pH bioflocculants and Kaolin particles absorb hydrogen ions (H^+^) and at high pH, hydroxide ions (OH^−^) are equally absorbed thereby interfering with the formation of bioflocculant-kaolin complex, thus leaving neutral pH as optimal for bioflocculation. Similarly, cations have been shown to neutralize negatively charged functional groups of both bioflocculant and suspended particles, thereby increasing the potential for adsorption of suspended particles to the bioflocculant thus enhancing flocculation [23]. Furthermore, the CPC purification of bioflocculant did not result in enhance flocculation activity; rather activity declined, thus suggesting that it is not cost effective to process the bioflocculants to CPB state. In addition, it is thought that the decline in activity of CPB is as a result of the use of metal ion surfactant (cetylpyridinium chloride) and subsequent dialysis, in its purification process, which rid the bioflocculant of metal ions to a large degree thus leaving PPB with higher cation content. This may have resulted in the higher flocculation activity observed in the PPB over CPB. In same vein, Cosa *et al.*, [14] and Wang, *et al.*, [24] showed that metal ions aid flocculation process.

### 2.7. Compositional Analyses of Purified Bioflocculant

Chemical analyses of the purified bioflocculant revealed presence of carbohydrates and proteins. However, detailed analyses showed that 100 mg of the purified bioflocculant yielded a total sugar content of 77.9 mg in the CPB and 76.3 mg in the PPB, while protein content was about 21.9 mg in the CPB and 22.6 mg in the PPB respectively. Further analyses of this carbohydrate component showed the presence of neutral sugars (35.88 mg and 32.94 mg), amino sugars (18.72 mg and 19.56 mg) and uronic acids (23.4 mg and 23 mg) for CPB and PPB respectively. These carbohydrate components were present in the percentage ratios 4.6:2.4:3 (CPB) and 4.3:2.6:3 (PPB) respectively. Conversely, CPC treatment (purification) did not alter the composition of the bioflocculant with respect to carbohydrates, proteins and other derivatives of CPB and PPB and this further confirms that the additional step in purification is unnecessary. The high uronic acid content of the bioflocculant makes it unique and promising for industrial application as bacterial polymers rich in uronic acids have been shown to ameliorate flocculation process [25,26].

### 2.8. FTIR Spectroscopy, SEM Micrography and Elemental Composition of Bioflocculant

**Figure 4:**
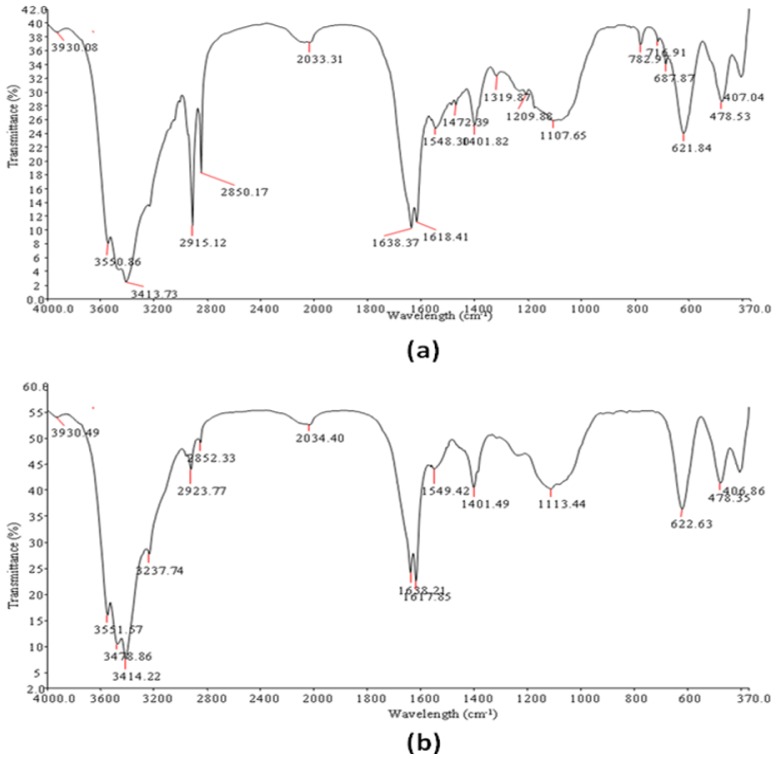
FTIR spectra of purified bioflocculant produced by *Streptomyces* sp. (Gansen) HQ537129 (**a**) partial purified bioflocculant (PPB) and (**b**) cetylpyridinium chloride (CPC) purified bioflocculant (CPB).

Fourier Transform Infrared spectra of the PPB and CPB (Table 3; Figure 4), displayed broad stretching peak at around 3551.57 to 3237.44 (cm^−1^) which is characteristic of hydroxyl groups from polymeric and dimeric OH stretch and a 1401.49 cm^−1^, typical of phenol or tertiary alcohol OH bend [27]. Similarly, wave numbers ranging from 2923.77 to 2852.33 (cm^−1^) is indicative of a weak C–H stretching band while 3378.86 cm^−1^ is indicative of aliphatic primary amine, N–H stretch. An asymmetrical stretching band at 1638.21 and 1549.42 (cm^−1^) and a weak symmetrical stretching band near 1401.82 cm^−1^ indicates the presence of carboxylic groups, carboxylate ions, an aromatic ring stretch and C–O and C–O–C from polysaccharides [28]. Likewise, 1107.65 cm^−1^ is indicative of other sugar derivatives while 622.63 and 478.35 cm^−1^ shows the presence of disulfides and aryl disulfide (S–S) stretch respectively [27,28].

**Figure 5:**
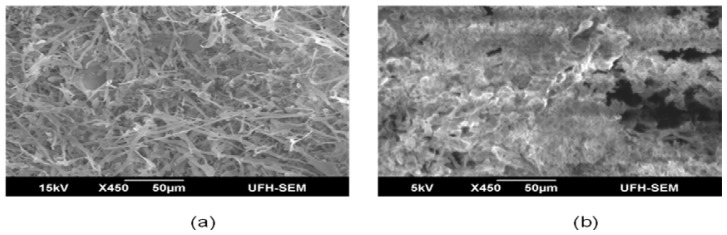
SEM Micrograph of purified bioflocculant from *Streptomyces* sp. Gansen (HQ537129). (**a**) CPB and (**b**) PPB.

Morphological elucidation of PPB and CPB via scanning electron micrograph shows a mesh like filamentous structure for CPB (Figure 5a), in marked difference from the PPB structure. Unlike the metal surfactant treated bioflocculants (CPB), PPB appeared to have a clump like structure with similarity to CPB only in the compact nature of the mesh (Figure 5b). Elemental analysis of PPB and CPB yields closely similar spectra with exception only in extent of peaks. These peaks are an indication of the quantity of the elements present. Quantification of these spectra (Table 4), showed PPB and CPB to have high ratios of Carbon (C), Nitrogen (N) and Oxygen (O). Sulfur and Phosphorus was detected in varying amounts. Furthermore, the variety of metal ions detected in PPB was more compared to CPB and this may have accounted for difference in structure as metal ions will cause PPB to be bound compactly. The presences of other elements as indicated by elemental analysis may be an indication that the bioflocculant is organo-metallic, or perhaps the metals could have originated from the fermentation medium. The FTIR spectrometry and elemental analysis gives better details of the presence of carbohydrates, proteins and possibly other components not assayed for as more functional groups were detected than have been accounted for.

### 2.9. Thermal Analyses of Purified Bioflocculant

**Figure 6:**
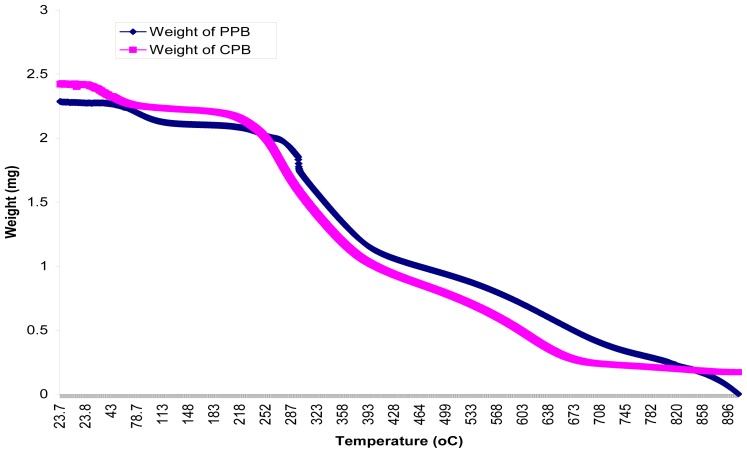
Pyrolysis curve of CBP and PPB through thermogravimetric analysis.

Thermal properties of PPB and CPB as studied by TGA, in the temperature range of 20 to 900 °C under nitrogen atmosphere, showed changes in content with decomposition. The mass loss of components is shown in the thermo-gravimetric plots (Figure 6). CPB and PPB started to decompose above 24 °C and 60 °C respectively. The thermo-gram for CPB and PPB exhibited three distinct decomposition steps which are similar to the pattern of decomposition for organic compounds documented by Niu *et al*. [29]. These steps were at 24 °C, 215 °C and 432 °C for CPB and 60 °C, 259 °C and 493 °C for PPB respectively. The first decomposition step of CPB stretched from 24 °C to 214 °C, while PPB stretched from 60 °C to 267 °C with corresponding weight loss of 0.4 mg and 0.5 mg respectively. Similarly, the second decomposition steps stretched up to 534 °C (CPB) and 567 °C (PPB) with weight loss of 1.9 mg and 1.8 mg respectively. This pyrolysis study suggests that the bioflocculant was basically composed of three compounds. This was validated via melting/decomposition temperatures which did not show a sharp point, but rather stretched over a range (results not shown), suggesting that the bioflocculant is composed of more than a single compound.

## 3. Experimental Section

### 3.1. Activation of Test Bacteria

An actinobacteria was previously isolated from water sample collected from Tyume river, Eastern Cape, South Africa, as part of the collections of the Applied and Environmental Microbiology Research Group (AEMREG), University of Fort Hare, South Africa. The bacteria was activated by inoculation into a sterile 5 mL broth composed of 3 g beef extract, 10 g tryptone and 5 g NaCl (per liter), and incubated aerobically overnight at 28 °C and 120 rpm.

### 3.2. Screening for Bioflocculant Production

The fermentation broth used in this study is the Basal salt media (BSM) composed as follows (g/L): glucose, 10; tryptone, 1; K_2_HPO_4_, 5; KH_2_PO_4_, 2 and MgSO_4_·7H_2_O, 0.3 [30]. A set of 500 mL flasks containing 200 mL fermentation medium (BSM) were aseptically inoculated with activated culture, by transferring 2% (4 mL) of the culture [15,16], adjusted to cell density of about 1.5 × 10^8^ cfu/mL [31] and incubating at a temperature of 28 °C for 72 h at140 rpm. After the incubation period, the fermentation broth was centrifuged (3000 rpm, 30 min, 15 °C) to separate the bacterial cells and the cell-free supernatant was assessed for flocculation activity.

### 3.3. Effect of Inoculum Size on Bioflocculant Production

Four flasks containing 200 mL fermentation medium were aseptically inoculated with 4 mL of the activated culture adjusted to cell densities of 1.0, 1.5, 3.0 and 5.0 (×10^8^ cfu/mL) respectively. These cultures were incubated at a temperature of 28 °C for 72 h at 140 rpm. After the incubation period, a portion of the fermentation broth was centrifuged (3000 rpm, 30 min, 15 °C) and the supernatant was assessed for flocculation activity. Other portion was used to determine bacterial load via viable cell count (cfu/mL) and turbidimetrically (OD_600_).

### 3.4. Measurement of Flocculation Activity

Flocculation activity was measured following the method of Zhang *et al*. [30] with slight modifications. Briefly, 0.3 mL of 1% CaCl_2_ and 0.2 mL of bioflocculant were added into 10 mL of Kaolin suspension (4.0 g/L) in a test tube. The mixture was vortexed using a vortex mixer (VM-1000, Digisystem) for 30 s and kept still for 5 min afterwards, 2 mL of the upper layer was carefully withdrawn and its optical density (OD) read spectrophotometrically (Helios Epsilon, USA) at 550 nm wavelength. A control experiment was conducted by repeating same process however; the bioflocculant broth was replaced with sterile (un-inoculated) fermentation medium. All assays were conducted in triplicates and the flocculation activity calculated using the following equations:

Flocculating activity={(A-B)/A}×100%

A and B are OD_550_ (optical density; 550 nm) of the control and sample, respectively.

### 3.5. Effect of Culture Conditions

Eleven flasks of 500 mL capacity, containing 200 mL fermentation medium were adjusted with 0.1 M NaOH and 0.1 M HCl to pH values corresponding from 2 to 12. Each flask was aseptically inoculated with activated culture (1.5 × 10^8^ cfu/mL) amounting to 2% of fermentation medium. These cultures were incubated at a temperature of 28 °C for 72 h at 140 rpm. After incubation, the fermentation broth was centrifuged (3000 rpm, 30 min, 15 °C) and the supernatant assessed for flocculation activity. The initial pH of the medium with optimum yield was repeated, but in incremental factor of 0.2 up to the next absolute value. After 72 h of incubation at 140 rpm the fermentation broth was centrifuged (3000 rpm, 30 min, 15 °C) and the supernatant assessed for flocculation activity. Similarly, the effects of incubation temperatures and agitation speed were assessed by varying incubation temperature by a unit of 2 from 24 to 38 °C while agitation speed varied by a unit of 40 from 120 to 400 rpm.

### 3.6. Effects of Carbon, Nitrogen and Cation sources on Bioflocculant Production

The BSM described in section 3.2 was used to assess the effects of carbon, nitrogen and cations on bioflocculant production, however, the sole carbon (glucose, fructose, sucrose, lactose, maltose and starch) and nitrogen (urea, ammonium sulfate, ammonium nitrate, ammonium chloride and peptone) sources were varied. Also, sole cation sources include monovalent salts (KCl and NaCl), divalent salt (MgSO_4_, CaSO_4_·H_2_O, MnCl·4H_2_O, and FeSO_4_) and trivalent salt (FeCl_3_). The fermentation conditions and assessment of culture supernatant for flocculation activity were same as in section 3.2.

### 3.7. Kinetics of Bioflocculant Production

A 200 mL fermentation medium inoculated with activated culture (1.5 × 10^8^ cfu/mL) amounting to 2% of the fermentation medium was incubated at 30 °C and 160 rpm and pH of 6.8 in a shaker incubator (based on optimum conditions determined previously). Bioflocculant production kinetics was monitored by withdrawing 5 mL of the fermentation broth; 4 mL was used for pH determination and assessment of flocculation activity, while the remaining 1 mL used to determine bacterial cell growth via viable cell count (cfu/mL). This process was conducted at 8 h interval for a period of 7 days.

### 3.8. Purification of Bioflocculant

Bioflocculant purification and concentration was done as described by Yokoi *et al*. [8] and Wu and Ye [32]. The fermentation broth was centrifuged (3000 rpm, 30 min, 15 °C) and cell pellets separated from the supernatant by decantation. Afterwards, the supernatant was mixed with ice cold ethanol (95%), at volume to volume ratio of 1:4 to precipitate the bioflocculant and kept at 4 °C in a cold cabinet for 16 h. The resulting precipitate was collected by centrifugation (10,000 rpm, 30 min, 15 °C) and re-dissolved in distilled water at ratio 1:4 (vol). This procedure was repeated twice and afterwards the partially purified bioflocculant (PPB) obtained was lyophilized. To another set, further purification, prior to lyophilization was carried out. The bioflocculant concentrate was dissolved in 0.05 M NaCl and 4 mL of 10% cetylpyridinium chloride (CPC) solution and the mixture stirred until the polysaccharide-CPC complex was completely solubilized. The mixture was left overnight at room temperature (~25 °C) after which the precipitate was recovered by centrifugation (10,000 rpm, 30 min, 15 °C). This process was repeated in two successive stages. Thereafter, the bioflocculant was re-dissolved in distilled water and dialyzed overnight against distilled water at 4 °C and the dialyzed polymer was re-precipitated with ice cold ethanol, followed by vacuum drying and lyophilisation [33]. The lyophilized fractions were designated as CPB and used for further flocculation analysis and characterization studies.

### 3.9. Effect of pH and Cations on Flocculation Activity

The flocculation activity of the partial and CPC purified bioflocculant was assessed using kaolin clay suspension method as described in section 3.4 above. However, 0.4 mg/mL of the purified bioflocculant was used in place of the bioflocculant free broth. The effect of pH on flocculation activity was determined by varying the pH of the kaolin clay suspension from 2 to 11. Likewise, the cation sources were varied from monovalent (NaCl and KCl) to divalent (CaSO_4_·H_2_O; MgCl_2_; MnCl·4H_2_O and FeSO_4_) and trivalent (FeCl_3_) metal ions, respectively. All other conditions for the flocculation assay remained the same.

### 3.10. Compositional Analyses of Bioflocculants

The total sugar and protein content of the purified bioflocculant were determined by the phenol–sulfuric acid method using glucose as the standard solution [34], and Folin-phenol method [35] using bovine serum albumen (BSA) as standard. Similarly, neutral sugar content was determined by the anthrone reaction [36] while amino sugars were determined in accordance with the methods of Elson-Morgan and Morgan-Elson, as seen in Chaplin and Kennedy [37] and lastly, uronic acids were determined using the carbazole-sulfuric acid method as described by Li *et al*. [26].

### 3.11. SEM Imaging, Elemental Analysis and FTIR Spectroscopy of Purified Bioflocculant

Bioflocculant samples placed on carbon coated stub were gold coated in a gold coating chamber, using Eiko IB.3 ION coater, thereafter; the scanning electron microscopic (SEM) image of the bioflocculant was obtained using JEOL JSM-6390LV FEI XL30 (JEOL; USA) scan electron microscope. The SEM was equipped with Noran Six 200 Energy Dispersive X-ray (EDX) Analyzer, and this was used to obtain the elemental composition of the bioflocculants. Similarly, the functional groups of these flocculants were analyzed using a Fourier transform infrared (FT-IR) spectrophotometer (2000 FTIRS Spectrometer; Perkin Elma System) over a wave number range of 4000 to 500 cm^−1^.

### 3.12. Thermal Studies of Purified Bioflocculant

Thermogravimetric analysis of the purified bioflocculants was carried out using a thermogravimetric analyzer (TGA 7; Perkin Elmer, Waltham, MA, USA) fitted with thermal analysis controller (TAC 7/DX). About 2–3 mg of partial and CPC purified bioflocculant, each was loaded into an alumina cup and weight changes recorded as a function of temperature for a 10 °C min^−1^ temperature gradient between 20 °C and 900 °C. A purge gas of flowing nitrogen at a rate of 20 mL min^−1^ was used. Likewise, melting point/decomposition temperature of the bioflocculant was determined using Gallenkamp melting point apparatus.

### 3.13. Identification of the Test Actinobacteria

The bioflocculant producing actinobacteria was identified via molecular technique as described by Cook and Mayers, [38]. The actinobacteria 16S rDNA gene was amplified by polymerase chain reaction (PCR) followed by sequence analysis of the amplified gene. The actinobacterial DNA was extracted through the boiling method and the PCR amplification was carried out in 50 μL reaction volume containing 2 mM MgCl_2_, 2 U Supertherm Taq polymerase, 150 mM of each dNTP, 0.5 mM of each primer (F1: 59-AGAGTTTGATCITGGCTCAG-39; I = inosine and primer R5: 59-ACGGITACCTTGTTACGAC TT-39) and 2 mL of the template DNA. Primer F1 and R5 binds to base positions 7–26 and 1496–1476 of the 16S rRNA gene of *Streptomyces ambofaciens* ATCC 23877, respectively [38]. The PCR condition included the following: initial denaturation (96 °C for 2 min), 30 cycles of denaturation (96 °C for 45 s), annealing (56 °C for 30 s) and extension (72 °C for 2 min), and a final extension (72 °C for 5 min). Gel electrophoresis of PCR products were conducted on 1% agarose gels to confirm that a fragment of the correct size had been amplified. Automated sequencing of the 16S rRNA genes of the bacterial isolates was performed using the Spectrumedix SCE2410 genetic analysis system with 24 capillaries. The sequencing reactions were performed according to the manufacturer’s instructions, using the Big Dye version 3.1 dye terminator cycle sequencing kit (Applied Biosystems) and 27F primer. The sequences were edited manually based on the most similar sequences.

## 4. Conclusions

*Streptomyces* sp. Gansen produced proteoglycan bioflocculant composed of uronic acids, neutral sugars, amino sugars and proteins with functional groups such as hydroxyl, carboxyl and amino groups favorable for flocculation. Bioflocculant production was optimal at log phase and optimum conditions include 2% (v/v) starter culture of 1.5 × 10^8^ cfu/mL, agitation speed of 160 rpm, pH of 6.8, and incubation temperature of 30 °C. Glucose, ammonium sulfate and magnesium chloride served as the best carbon, nitrogen and cation source for bioflocculant production. These results suggest promising application of these bioflocculants as they effectively flocculated kaolin clay in aqueous media.

## Figures and Tables

**Table 1:** The effects of carbon, nitrogen and cations sources for the production of bioflocculant by *Streptomyces* sp. Gansen (HQ537129).

Carbon Source	Glucose	Lactose	Fructose	Sucrose	Maltose	Starch	
Max. Flocculation activity (%)	89.26	72.54	86.41	59.33	43.0	38.9	
Bioflocculant yield (g/L)	3.37 ± 0.22	2.91 ± 0.40	3.4 ± 0.17	2.7 ± 0.13	1.95 ± 0.37	1.52 ± 0.31	
**Nitrogen Source**	**Urea**	**(NH_4_)_2_SO_4_**	**(NH_4_)_2_NO_3_**	**(NH_4_)_2_Cl_4_**	**Peptone**		
Max. Flocculation activity (%)	69.18	76.27	53.45	40.18	65.63		
Bioflocculant yield (g/L)	1.72 ± 0.09	3.26 ± 0.81	2.51 ± 0.19	1.84 ± 0.26	2.93 ± 0.31		
**Cation Source**	**KCl**	**NaCl**	**MgCl_2_**	**CaSO_4_·H_2_O**	**MnCl·4H_2_O**	**FeSO_4_**	**FeCl_3_**
Max. Flocculation activity (%)	34.66	29.58	73.19	61.74	46.19	33.18	21.89
Bioflocculant yield (g/L)	1.64 ± 0.33	1.26 ± 0.52	3.39 ± 0.05	2.71 ± 0.38	2.64 ± 0.71	1.85 ± 0.51	0.98 ± 0.31

**Table 2:** The effects of cations and medium pH on the flocculation activity of purified bioflocculant produced by *Streptomyces* sp. Gansen (HQ537129).

Bioflocculant	Cation Sources/Flocculation Rate (%)									

	Na^+^		K^+^	Mg^2+^	Mn^2+^	Ca^2+^	Fe^2+^		Fe^3+^	
	
**PPB**	56 ± 1.4		49± 2.2	76 ± 0.9	62 ± 1.6	87 ± 1.2	47 ± 1.1		51 ± 1.9	
**CPB**	32 ± 0.8		40 ± 1.7	57 ± 2.2	54 ± 0.7	72 ± 1.9	42 ± 0.5		46 ± 1.3	

	**pH/Flocculation Rate (%)**									
	
	2	3	4	5	6	7	8	9	10	11
	
**PPB**	0	14 ± 2.5	29 ± 1.8	38.5 ± 2.4	49 ± 2.6	87.7 ± 1.9	70.7 ± 2.2	67 ± 3.1	64 ± 2.7	36 ± 2.3
**CPB**	0	0	11 ± 0.8	27 ± 1.2	39 ± 0.6	76.9 ± 2.3	58.4 ± 1.8	52 ± 2.2	45 ± 1.7	19 ± 1.5

**Table 3:** Position and characteristic bond obtained from Fourier Transform Infrared Spectroscopy of bioflocculants from *Streptomyces* sp. Gansen (HQ537129).

Compound	Origin	Group Frequency Wave Number (cm^−1^)			Assignment/Functional Group

		Assigned	CPB	PPB	
Hydroxy and ether compounds	O–H	3570–3200 (broad)	3551.57; 3414.22; 3378.86; 3237.44	3550.86; 3413.73	Hydroxy group, H-bonded OH stretch

Amino compounds and Polysaccharides	O–H	3400–3200	-	-	Normal “polymeric” OH stretch

	O–H	3550–3450	-	-	Dimeric OH stretch

	O–H	1410–1310	1401.49	1401.82	Phenol or tertiary alcohol, OH bend

	N–H	3400–3380	3378.86	3413.73	Aliphatic primary amine, N–H stretch

	N–H	3510–3460	3551.57	-	Aromatic primary amine, N–H stretch

	>N–H >C=O C–O C–H	1650–1550	1638.21; 1617.85; 1549.42	1638.37; 1618.41; 1548.30	Secondary amine, NH bend associated with proteins, >C=O stretch, Ether, Carboxylic groups, C–H bend from from CH_2_, C–O bend from carboxylate ions C–O, and C–O–C from polysaccharides

Methyl (–CH3)	–CH	2935–2915/2865–2845	2923.77; 2852.33	2915.12; 2850.17;	Methylene C–H asym./sym. stretch
	>CH–	2900–2880	2852.33	2850.17	Methyne C–H stretch (Methyne)

Aromatic ring (aryl)	C=C–C	1510–1450	1549.42	1548.30	Aromatic ring stretch

Thiols and thio-substituted compounds	S–S	620–600	622.63	621.84	Disulfides (S–S stretch)
	S–S	500–430	478.35	478.35	Aryl disulfides (S–S stretch)

CPB = wave number (cm^−1^) obtained for CPC purified bioflocculant; PPB = wave number (cm^−1^) obtained for partial purified bioflocculant.

**Table 4:** Quantification of the elemental composition of bioflocculant produced by *Streptomyces* sp. Gansen (HQ537129).

Partial Purified Bioflocculant						

Element Line	Element Wt.%	Wt.% Error	Atom %	Atom % Error	Compound Formula	Compound Wt.%
C K	24.82	+/−1.05	48.22	+/−2.04	C	24.82
N K	7.14	+/−1.36	11.89	+/−2.27	N	7.14
O K	20.61	+/−0.50	30.06	+/−0.72	O	20.61
Na K	0.25	+/−0.06	0.26	+/−0.06	Na	0.25
Mg K	0.63	+/−0.04	0.60	+/−0.04	Mg	0.63
Al K	0.14	+/−0.04	0.12	+/−0.03	Al	0.14
P K	2.84	+/−0.12	2.14	+/−0.09	P	2.84
S K	1.06	+/−0.10	0.77	+/−0.07	S	1.06
K K	0.57	+/−0.05	0.34	+/−0.03	K	0.57
Ca K	0.32	+/−0.05	0.19	+/−0.03	Ca	0.32
Cu K	1.92	+/−0.30	0.71	+/−0.11	Cu	1.92
In L	0.00	+/−0.00	0.00	---	In	0.00
Au L	39.69	+/−3.40	4.70	+/−0.40	Au	39.69

**CPC Purified Bioflocculant**						

C K	38.09	+/−0.74	64.23	+/−1.24	C	38.09
N K	3.38	+/−1.28	1.86	+/−2.29	N	3.38
O K	15.95	+/−0.72	20.19	+/−0.91	O	15.95
Na K	3.21	+/−0.14	2.83	+/−0.13	Na	3.21
Al K	0.40	+/−0.05	0.30	+/−0.04	Al	0.40
P K	1.24	+/−0.11	0.81	+/−0.07	P	1.24
S K	0.62	+/−0.15	0.39	+/−0.09	S	0.62
Cl K	8.10	+/−0.22	5.77	+/−0.13	Cl	8.10
K K	0.33	+/−0.07	0.17	+/−0.04	K	0.33
Cu K	2.30	+/−0.45	0.73	+/−0.14	Cu	2.30
Au L	26.47	+/−4.76	2.72	+/−0.49	Au	26.47
